# Use of Universal Design for Learning Principles in a Public Health Course

**DOI:** 10.5334/aogh.4045

**Published:** 2023-07-21

**Authors:** Tara Casebolt, Kimberly Humphrey

**Affiliations:** 1 Boston College in the Global Public Health and Common Good Program in Chestnut Hill, MA, USA; 2 Center for Teaching Excellence at Boston College in Chestnut Hill, MA, USA

**Keywords:** Universal design for learning, pedagogy, teaching, public health education

## Abstract

**Background:**

Universal Design in Learning (UDL) is a pedagogical framework that increases accessibility in the classroom for students by offering structured flexibility regarding coursework. The main tenets of UDL are to: 1. provide multiple means of engagement; 2. provide multiple means of representation; and 3. provide multiple means of action and expression.

**Objective:**

This study aims to determine if students will use the extra options inherent in UDL if offered and if they are satisfied with the course that uses UDL.

**Methods:**

This study evaluates a maternal health course for first-year students that was designed using UDL principles and taught at Boston College in the spring semester of the 2021–22 academic year. The course included 26 students. Surveys and a focus group were used to gather both qualitative and quantitative data.

**Findings:**

All technological tools and learning options offered were used by at least some of the students. The majority of students submitted assignments via alternative format options and used non-traditional learning materials like podcasts and videos. Students rated their satisfaction with the course highly. During the focus group, students expressed that they appreciated the increased flexibility of having multiple ways to learn and show the knowledge they had acquired.

**Conclusions:**

UDL can work in an undergraduate-level public health course. Students learn well and are satisfied with courses when UDL is used for course design. Additional research needs to be done to determine if learning outcomes are impacted by the use of UDL and if UDL could be used in graduate-level public health courses as well.

## Introduction

According to CAST—originally called the Center for Applied Special Technology, but now only go by CAST—an expert organization in the field of Universal Design for Learning (UDL), UDL “is a framework to improve and optimize teaching and learning for all people, based on scientific insights into how humans learn” [1]. UDL is implemented in the classroom using a series of guidelines, creating a framework through which practical strategies for infusing UDL into a course can be accomplished. The three primary UDL Guidelines are: 1. provide multiple means of engagement; 2. provide multiple means of representation; and 3. provide multiple means of action and expression [2].

Studies have previously been conducted regarding the use of UDL in college courses. These studies have found that the structured flexibility inherent in UDL—particularly regarding having multiple, clear options for assessment, participating in the course, and learning course content—has a positive impact on students’ comprehension of the course content. Using the UDL Guidelines are also an efficient way to implement this flexibility [3]. The use of a UDL framework when developing a class has also been shown to make the goals of the course clearer to students, in addition to increasing the engagement of students on the course [4]. Because of its flexibility, the use of UDL has also been shown to increase courses and instructors’ ability to meet the learning needs of a variety of diverse students on a single course [5].

There has been particular interest in using UDL to create a classroom that is more accessible for students with disabilities, eliminating barriers to participation for these students [6,7]. Application of the UDL Guidelines to a course can have a positive impact on all students, regardless of ability status, though critical disability scholars argue that focusing on this universal benefit undermines the prioritization of the needs of students with disabilities [8,9]. Implementing UDL in a classroom for the benefit of all students should not replace the accommodation of disabled students’ needs. There has been limited research into the effect of UDL in the public health classroom, and research needs to be conducted to determine if the findings regarding UDL’s impact in other post-secondary courses also applies to public health courses. This article is focused on a public health course for two main reasons. The first is that one of the authors is a professor of public health. The second is that there has been pushback regarding the inclusion of students with disabilities, learning differences, or those from other marginalized groups in health education. Some argue that adapting this field for those outside what is considered a “traditional” student, i.e. an able-bodied student who does not need accommodations, would harm the quality of education pupils receive. There are also suggestions that allowing students who have disabilities or learn differently to participate in health education would harm patients or the public [10]. This is not the case, and there are detriments to excluding these populations in health education programs. The use of UDL could counteract some of these issues and therefore should be applied in health education, including public health training.

This study aims to address the following research questions: Do students in public health courses make use of the resources available in a class implementing UDL practices and principles? What additional resources are used most by students? And are students satisfied with a course that uses UDL principles in its design?

## Methods

### Study setting

To conduct this study, the authors used a convenience sample of students enrolled in a maternal health course taught by the first author. The course used for this study was specifically for first-year university students. There were 26 students enrolled in the course. This course was part of the core program at Boston College, which is a unique educational structure that allows students to fulfill general education requirements through more interesting, interdisciplinary courses. This course was paired with another about neuroscience and maternity, including discussion of child development. Students had to be enrolled in both courses simultaneously. The courses are not required. Students must take a specific number of core course, but can select which ones they take. There were no prerequisites for the course, and students of any major could enroll. A combination of lectures, small group activities, large group discussions, and out-of-classroom experiences were used. Some learning objectives for the class included: “students will engage with educational materials from a variety of social science fields, like public health, anthropology, and sociology, to better understand the maternal experience”; students will engage critically with past and present instances of injustice via the discussion of the history of birth and motherhood and the unique experiences of marginalized communities, racial minorities, people living in poverty, LGBTQ+ parents, parents with disabilities, etc.”; and “students will apply social science methods to complete assignments focused on expanding their knowledge regarding society’s effect on mothers and parents.”

### Course design

The course was designed to implement UDL principles in a variety of ways. First, all materials for the course were provided in a variety of formats, including journal articles, videos, podcasts, white papers, organization reports, and blogs. The instructor selected these materials to ensure they covered the same content, but through a variety of methods of communication and levels of specificity. Students were able to select which resources they would focus on for class preparation. This ensured they were able to learn the relevant information in the ways that worked best for their learning and information processing abilities. They could also focus on materials written at a level they could comprehend this early in their higher education career. Students in the class came from a variety of high school backgrounds, and all had different levels of training to read scientific literature. Providing the materials in many formats made it possible for students with less experience of scientific literature to learn the same information as their peers, but in a way they would be able to understand.

Secondly, students had a variety of options when completing assignments. All assignments had specific learning goals for pupils to achieve, but students could select from multiple submission formats. These formats included written papers, video essays, podcast episodes, or scientific posters. Thirdly, participation in the course was evaluated not only through traditional definitions, such as speaking in large class discussions, but also via small group work in class, asking questions during class through online polling, posting questions on an online discussion board, and attending class each week in person or via Zoom. Finally, all materials provided during the course were accessible for students who used assistive technology or had information processing needs. For example:

- videos had captions or transcripts;
- podcasts had transcripts;
- documents were compatible with screen reader technology;
- slides were provided before class so they could be loaded into screen reader software as needed;
- all images contained alt text;
- documents and slides were run; and
- through a filter to determine accessibility for color blindness.

Students were informed about accessibility assistance options, such as speech-to-text and text-to-speech software, color changing options for materials, spell check, magnifiers, alt text for images, etc. so they would be able to use these tools as needed. As a result of these elements, the course met the three UDL guideline principles.

Students were given the option to not participate in the study, and 24 students elected to participate. They were also told that study participation would not impact their grade on the course. All data analysis occurred after the end of the semester, when the course was taught, to ensure the data from the study would have no impact on students’ grades. The focus group was run by the second author, who was not involved in grading the students or teaching the course. The transcripts were created by a graduate student not involved in grading the class or teaching the course, and the transcript was deidentified before the first author analyzed it. The study was evaluated by the Institutional Review Board at the university and was granted an exception as a minimal risk study. However, anonymous consent was collected for both the survey (via a question at the survey’s beginning) and for the focus group (with anonymous consent forms). Anonymous consent means that the students would either put a checkmark on paper forms consenting to the focus group or would click a checkbox consenting to the survey, as opposed to signing their names. This means that the first author did not know who participated.

### Data collection

This study used both qualitative and quantitative data to address the research questions. Data was collected via two methods. Quantitative data was collected via an anonymous online survey through Qualtrics; this was sent to the students enrolled on the course at the end of the semester. The survey data was not accessed by the authors until after grades were due for the semester, so fear of responses affecting their grades would not impact students’ responses to the questions. This survey included questions based on four themes: classroom environment, opinions about class choices, use of technology, and use of learning options provided (i.e., attendance, learning materials, and assignments). To assess the use of technology and learning options, students were presented with a list of these tools and options and were asked if they used them very often, often, sometimes, or never. For the environment and opinions questions, students were provided with a list of statements and were asked if they strongly agreed, somewhat agreed, neither agreed or disagreed, somewhat disagreed, or strongly disagreed. A focus group was conducted with the students during the last week of the semester to capture additional qualitative data that cannot be collected via a quantitative survey.

### Data analysis

The quantitative data from the Qualtrics survey was analyzed in Microsoft Excel. Descriptive statistics, including counts and percentages, were calculated to determine the most common responses for each question. The transcript from the focus group was analyzed in Atlas.ti. Themes were identified, and the transcript was coded according to those themes. All themes were identified via emergent analysis and no a priori codes were used. Only the first author coded the focus group transcript.

## Results

### Survey responses

**Figure 1:**
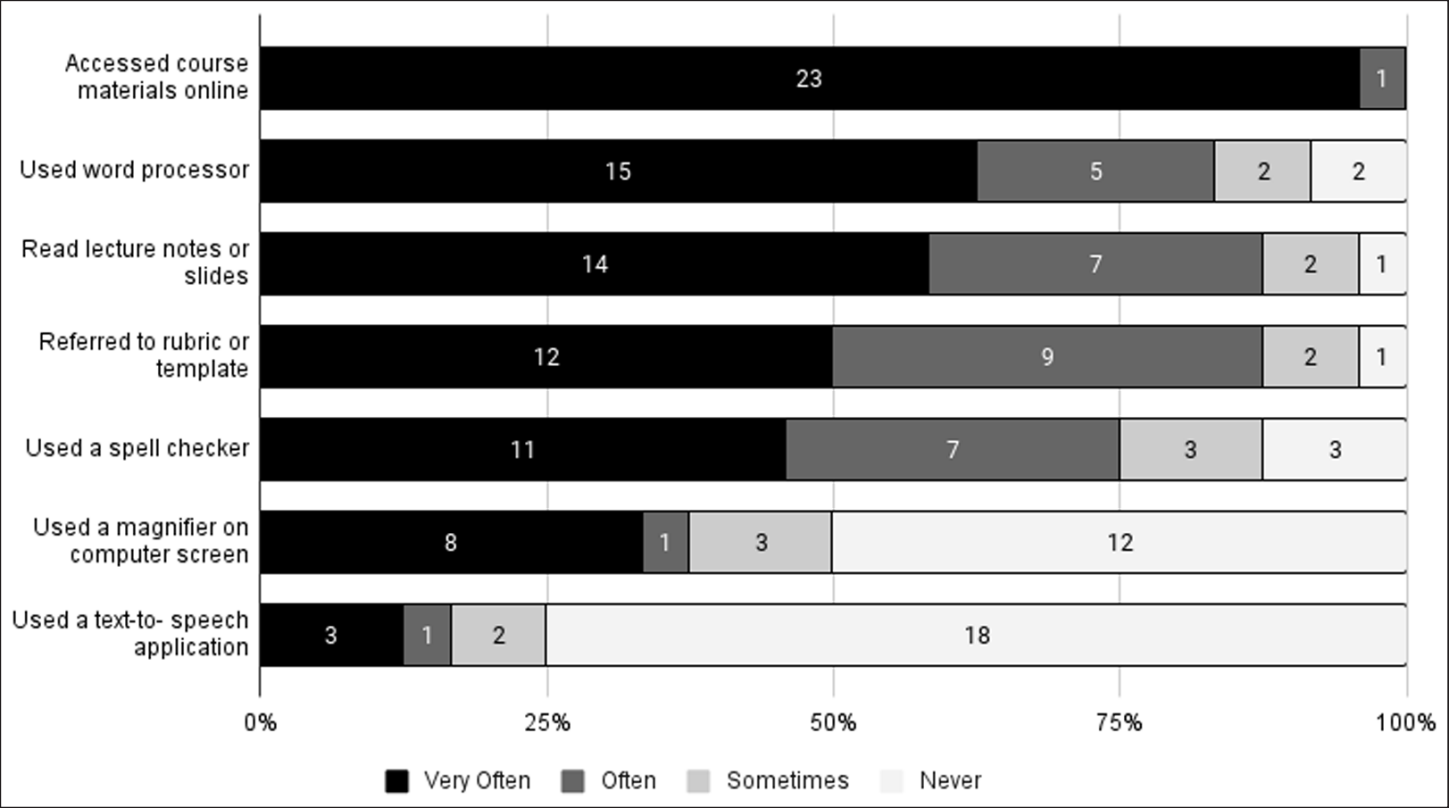
Counts of Reported Use of Technology by Participants (n = 24).

All students in the class reported using at least one of the technological tools offered for the class, and all tools were used by at least one student. The majority of students reported: using the course website to access materials; reading lectures notes or slides provided by the professor; using a word processor to create assignments; and using a provided rubric or template when developing assignments. Only a couple of students reported using speech-to-text or text-to-speech applications, or changing the background colors of their screens to aid in viewing course materials. This is not surprising, as these are tools most commonly used by individuals with specific information processing or visual disabilities. However, it is important to note that none of the students in the class had official accommodations related to these tools or the types of disabilities most related to these tools. This suggests that some students benefit from these tools even if they do not have an accommodation requiring their availability. See Figure 1 for more details.

**Figure 2:**
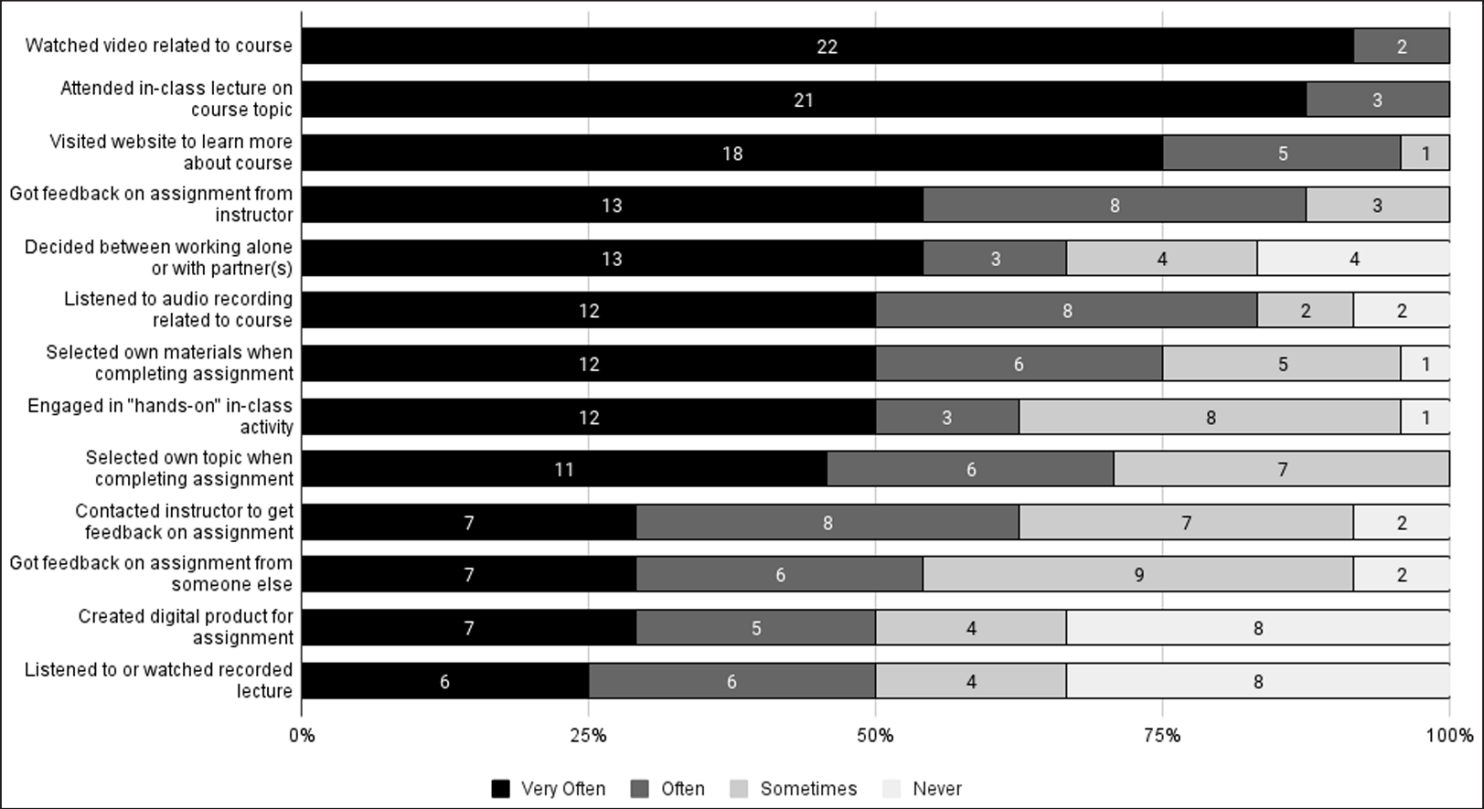
Counts of Reported Use of Learning Options (n = 24).

The majority of students in the class reported utilizing all of the learning options offered in the course. Students were given a choice of formats to view course materials when preparing for each class. From session to session, these could include readings from articles or books, website-based materials, videos, podcasts, or news clips. All students reported reading written materials, using web-based materials, and watching videos, while 92% used audio preparatory materials. All students attended classes in person, and 67% used recorded lectures when unable to attend in person. For the assignments, all students selected their own topics for assignments and received feedback from the professor; 96% selected all of their own materials for their assignments; 92% specifically reached out to the professor requesting feedback on assignments; and 92% received feedback from someone else on assignments. See Figure 2 for more details.

**Figure 3:**
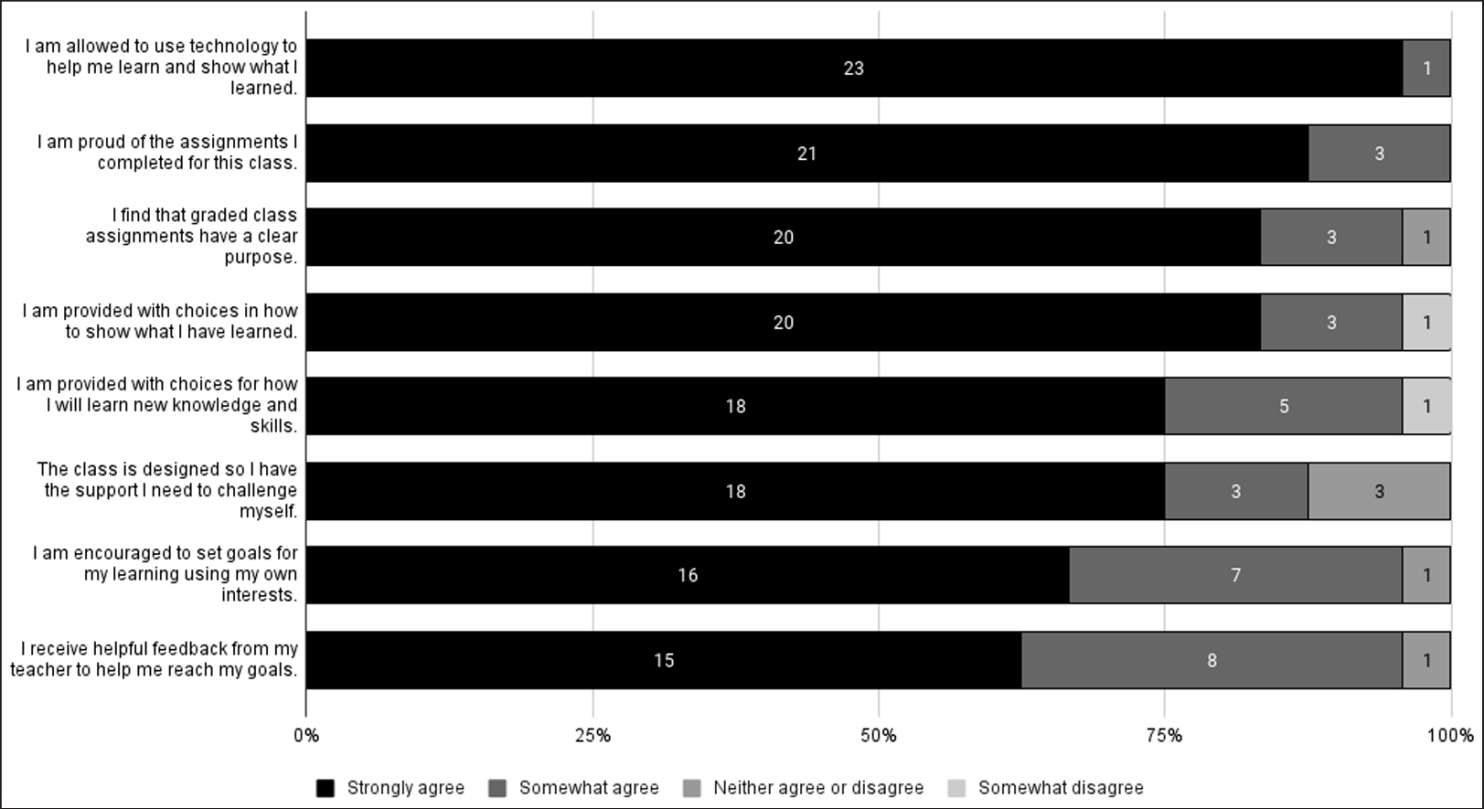
Counts of Opinions About Class Choices (n = 24).

Having the choice to ask for help and access to needed assistance is an important element of UDL. When asked whether they felt they were given adequate choices and access to assistance as needed, all students agreed that they were allowed to use tools and technology to help them learn and show what they learned, and that they were proud of the assignments they completed for the class. The majority of students (96%) stated that they were provided with choices for how they would learn new knowledge and skills; they were provided with choices to show what they learned; their graded assignments had a clear purpose; they received helpful feedback and tips from their teacher to help reach their goals; and that they were encouraged to set learning goals using their own interests. In general, these students felt the class provided sufficient choices to enhance their learning experiences. See Figure 3 for more details.

**Figure 4:**
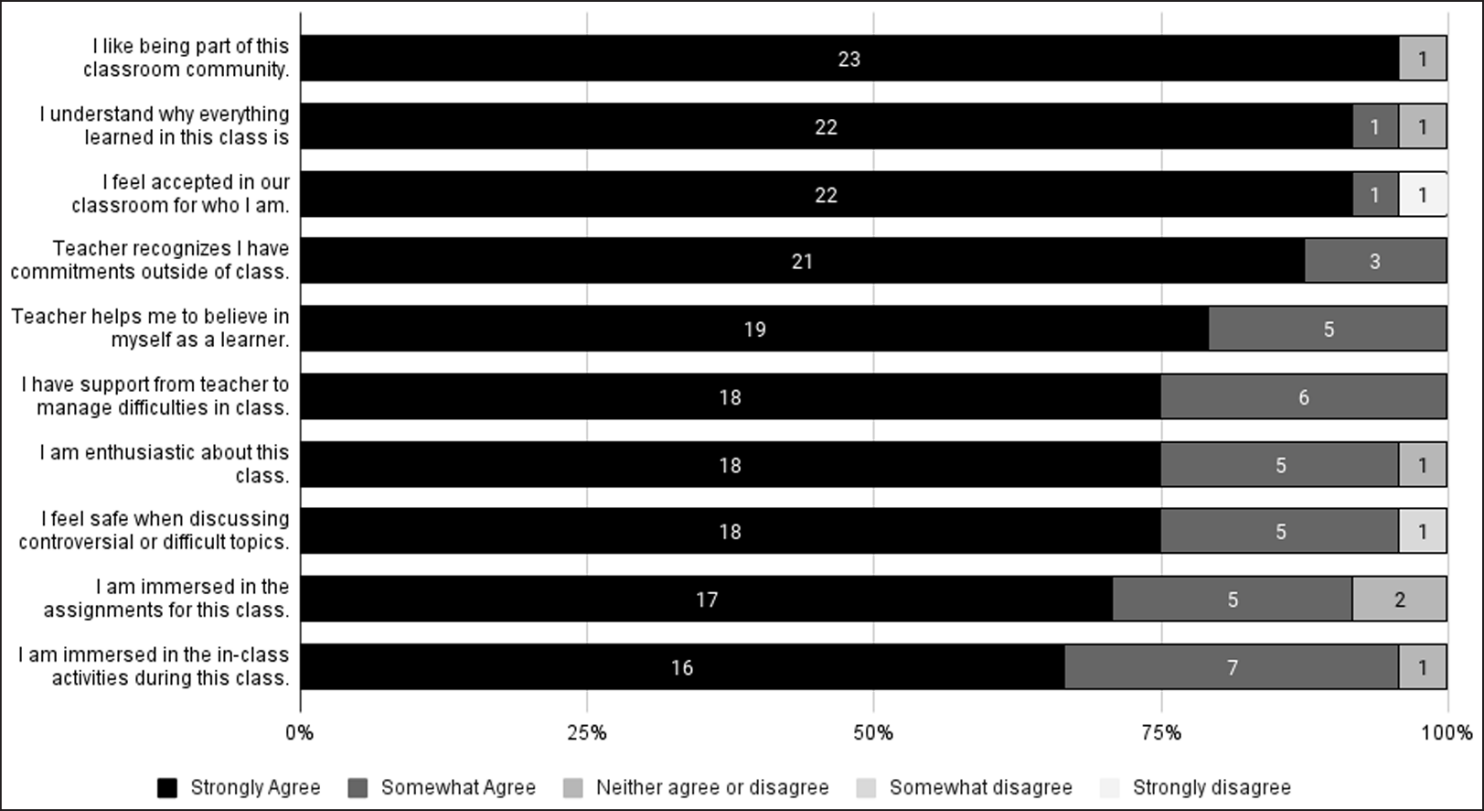
Counts of Reported Feelings About Classroom Environment (n = 24).

Students also had positive feelings about their experience of the class overall. All students reported that their teacher recognized that they had commitments outside of class, helped them manage difficulties, and helped them believe in themselves as learners. These elements demonstrate that the course and the professor considered the whole person and students’ experiences—setting reasonable expectations within the context of university norms, while also providing the flexibility to work around emergencies or the natural difficulties of being a first-year university student. In addition, 96% stated that they liked being part of the classroom community; understood why what they were learning was important; were enthusiastic about the class; felt accepted in the classroom for who they were; felt safe discussing difficult topics in class; and were immersed in in-class activities. Overall, the classroom environment was a positive one. See Figure 4 for more details.

### Focus group

The following themes were identified during the analysis of the focus group: accessibility; assignment feedback; assignment options; class organization; clearer connections; enjoyment of class; flexibility; inaccessibility; material options; need of more guidance; need of more diversity; positive classroom environment; and real-life application. Many of these codes referred to positive elements of the course design, while a few discussed negative experiences with the class or suggestions for improvements to it.

There were four main codes that reflected positive comments about the course: assignment options, class organization, flexibility, and positive classroom environment. When discussing the assignment options, students agreed that being able to choose the topic and format of their assignments had a positive impact on their learning experience. For example, one student stated: “…there was always multiple options of fulfilling your requirements … always gave like multiple options for what we wanted to do, and there was also like some sort of choice in what you wanted to research or look into…” This quote is indicative of the feelings of most students who commented on the choices they had when developing and submitting assignments.

Comments regarding flexibility referred to a number of course elements. One was attendance options, particularly the option to attend the course via Zoom. While this course was taught in the Spring semester of the 2021–22 school year (January through May of 2022), when most colleges and universities in the United States had returned to in-person classes, there were still COVID cases among the students in the course, and additional students experienced other illnesses and injuries throughout the semester. A student who experienced illness during the semester stated: “having … Zoom class available, I know like I had COVID and obviously couldn’t come into class … but having the Zoom option and recordings available and being available to talk over Zoom was super helpful”. Another element of flexibility was the variety of learning materials offered, increasing the accessibility of the course. One student stated: “…we all learn in different ways and … rather than just heavy scientific readings, she [the professor] offers podcasts and videos and YouTube videos, or movies and other ways to have us interact with the material.”

Organization of the course was something many students commented on during the focus group, with most comments focusing on the use of the online learning platform, Canvas, and providing detailed information on assignments from early in the semester. In general, the students appreciated how much information was available on Canvas and the clarity of the website layout. For example, one student said: “I have to agree about Canvas—the organization of Canvas was really nice. I know that in other classes, half the time professors don’t even know where to put things. They’re like, ‘Oh let me search to see where I put this,’ which is really difficult when they can’t even find documents or things that we need, whereas this was, like we didn’t ever have to look at the syllabus. Everything was in order. It was very organized, everything for that topic was under the same thing, like the same umbrella, and so it wasn’t like ‘It’s in this. Oh wait, no I’m wrong. It’s in this. Maybe it’s in this.’ And so, it made it a lot easier to follow along and find things, rather than when professors can’t even find where things go.” Another positive element of the course organization, as highlighted by students, was the availability of assignment descriptions and details early in the semester, particularly in the form of rubrics for each assignment and each submission method option. For example, for a single assignment there would be separate rubrics for a paper, a video, or a podcast so the students knew the expectations for each submission method option. When discussing this, a student reflected: “…she [the professor] uploaded the rubrics for our big projects, which were a very large percentage of our grade, and I thought…having those accessible to us at the very beginning of the year, like what was expected of us…it’s like ‘Oh, okay let’s do this’.”

Creating a positive classroom environment was a key goal for this course, particularly as the course covered a number of topics that can be considered controversial or difficult to discuss, like abortion, family planning, gender-based violence, etc. Several students mentioned this specifically during the focus group, most agreeing that this goal was achieved during discussions in the course. One student explained this feeling in this way: “I was just gonna say that she [the professor] was able to talk about very controversial topics and current events and like, in a non-biased point of view without asserting too much opinion, so regardless of what anyone feels on that topic, she would make everyone feel comfortable.” There appeared to be a general consensus that part of the reason students felt comfortable discussing difficult topics was the availability of multiple participation methods. If students wanted to make a point they thought would be controversial, they could do so anonymously via polls, which were often used when contentious topics were discussed. Also, students expressed feeling more comfortable when discussing in small groups. as opposed to the whole classroom. Discussions of controversial or political topics in courses can lead to concerns about faculty being biased towards one argument or point of view. Because of these concerns, some students would argue that professors should stay completely neutral on these topics, removing the potential impact of their biases. While the issue of faculty bias and whether or not it is a goal faculty should have is hotly debated, it is something some students believe is important and will include when judging the effectiveness of their professors [11,12].

There were also some negative aspects of the course, related to either its design or the course experience, as expressed by students. The most common one was a need for additional guidance. Providing more options means that students also need more information on how to implement these, particularly as this course was specifically for first-year students in their second semester. These students were still adjusting to college courses in general, and therefore, when presented with more flexibility, they needed more information suggesting how to take advantage of these options. In particular, students mentioned needing more structure for their final project, a large research project they worked on throughout the semester. One student said: “I don’t know if this is just a personal thing, but I know there was a timeline for our final project that I didn’t really end up using … And for the other class, there were like specific dates that the professor wanted drafts of our work. I think that was nice because it made it helpful, and I knew this was there, but since there was no pressure to like, turn something in, I don’t know if I feel like I will be hard-pressed for time in my writing. That might just be me, but maybe being more intentional about putting this timeline into place might help.” It is important to note that the student was referring to the neuroscience and maternity course mentioned above when they said “the other course.” Students expressed a preference for required deadlines as opposed to suggested guidance. Providing guidance as opposed to deadlines was a choice the course instructor made to maximize flexibility, but as UDL advocates for structured flexibility, this element of the course leaned too heavily on flexibility and not structure. This suggestion was applied to future iterations of this course.

Two other concerns expressed by students regarding the course were the inaccessibility of materials and a lack of diversity. One student stated that the course slides were too dense and needed to be simplified to help students process and understand the information better. This was the main accessibility concern. Slides were mainly used to help reinforce points from the readings and guide in-class activities, but changes have been made to slides for future iterations of the course to reflect this comment. There were also a couple of comments about the diversity of the student make-up of the course. Over 90% of the students in the course were cisgender female, limiting the perspectives available during course discussions. In addition, approximately 80% of the students were white and most students were from middle- or upper-class economic backgrounds. This led to even more limitations in perspectives. Students suggested that the course would have been better if there had been a more diverse student body. See Supplemental Table 1 for all qualitative themes and a sample quote for each of those themes.

## Discussion

Based on the focus group and survey, it is clear that students felt they could choose how to learn and present their new knowledge, and believed these choices had a positive impact on their course experience. Even though students did not know what UDL was and were not informed about the professor’s intentions to use UDL to increase accessibility, they were able to identify some of the key components of UDL used in the course. The majority of students agreed they were given options in all elements of the course, and in the focus group, students expressed that these choices improved their experience. Many students took advantage of the different options for assignment submission, using the course as an opportunity to express their learning in a different way or to try a new modality when creating assignments. While students in this course did not have accommodations requiring accessibility tools or more accessible learning options, when these tools were offered, the students used them. Every accessible tool offered was used by at least one student. Students took advantage of the different formats available to support their learning, and this helped them approach the course topics through a multitude of different lenses.

The concerns regarding the make-up of the student body of the course is largely a result of the demographics of the university where the course was taught. The majority of students at the university are white and middle- and upper-class, so the students enrolled in the course will reflect this. However, for future semesters the professor and their supervisors have made an attempt to recruit students from under-represented student groups. The professor has also worked with supervisors to increase male enrollment in the course, including considering a change in the course name and description. However, it is important to note the majority of students in public health, medical, and nursing programs are female, and courses in these fields attract more female students overall [13].

One argument against the use of UDL in college courses is that it is too time consuming or resource intensive on the part of the faculty member. The need to update course materials, develop new assignments, include new technology, etc., is seen as burdensome and a barrier to implementing UDL. There are also some faculty members concerned with whether or not using UDL is fair for all students, as some see it as catering to the needs of the few [14]. The feeling of being overwhelmed, unprepared, or uncomfortable with implementing UDL on the part of professors can be overcome through education about the UDL principles and strategies for their use in the classroom. One study found that the more knowledge faculty reported about UDL, the more likely they were to have a positive attitude towards UDL and feel comfortable with implementing its principles on their courses [15]. Another determined that after faculty attended UDL workshops, they were more invested in using the UDL model and more strongly believed UDL could help their students engage with even more materials on the course [16]. Faculty at some institutions are actively asking for and seeking additional education and professional development in UDL so they can better implement it [17].

Undergraduate students are generally supportive of faculty using UDL in their courses. A study in Canada found that students reported many of the UDL elements useful in assisting learning and used most of the options and materials provided to them by professors [18]. Another study in Tennessee found that students were more motivated to participate in courses if they used UDL [19]. Students of a health sciences class using UDL reported feeling that they had more flexibility, less stress, and greater empowerment because the course followed UDL principles [20]. This provides additional evidence that UDL can work in health-focused courses.

Use of UDL is often discussed within the context of assisting students with disabilities to better participate in the classroom and learn effectively [14,21,22]. However, the results of this study provide evidence that using UDL can have a positive impact on all students, not only those with disclosed disabilities or those with registered accommodations. This is particularly important for students from disadvantaged backgrounds who might not have an official diagnosis or the paperwork required for accommodations because of a lack of access to healthcare and testing services. Also, a recent study from the National Center for Education Statistics found that the majority of students with disabilities were not using accommodations or requesting them, often fearing stigma or needing independence [23]. Faculty can never be completely sure that there are no students with disabilities or other learning needs in their classroom who could have a more positive experience if UDL principles were applied in the classroom.

### Limitations

There are some limitations to this study. First, it is based on a case study of a single course with a small sample size. It was also conducted at a private institution with limited diversity among the student body. Therefore, the findings of the study are not generalizable. There is no ability to conduct a comparison to another cohort of students, as this was the first time the course was taught, and there is no guarantee the course will be taught again. Students were not assessed about their attitudes at the beginning of the course. This was done intentionally to reduce potential bias but is a limitation to the study design, as it does not allow for pre- and post-intervention analysis. All students in the course were first-year students. This could be a strength or limitation, as they did not have a great deal of previous experience with college courses, which could reduce bias or could also lead to students having inaccurate expectations for college courses based on their experiences in high school.

### Pedagogical and research recommendations

UDL has been shown to be effective in undergraduate courses. Students are more satisfied with courses when UDL is used, feeling they have more flexibility, control over their learning, and ability to show what they have learned [24]. This is something that can be implemented well in health courses. More health professionals and scientists are using alternative methods to communicate information to the public, increasing options to provide learning materials to students [25,26]. The use of these other educational methods also implies a need to train public health personnel on these communication strategies. Allowing students to present their research using an alternative method increases the communication skillset of the future public health workforce. Using UDL will not only make public health courses more accessible to students with disabilities and those with official accommodations, but to all students regardless of their disability status. UDL can create a more equal playing field in education, providing students with a greater opportunity to succeed. Based on all of these arguments, UDL should be included in the design and teaching of undergraduate public health courses. Public health faculty should receive training on UDL and be provided with the resources and professional development necessary to create more accessible, inclusive public health courses for all of their students. More research needs to be done to determine if learning outcomes are impacted by the use of UDL by comparing the assignment quality, test scores, and knowledge retention in traditional, lecture courses with that of courses using UDL principles. Also, studies should be conducted to determine if UDL would be effective in graduate-level public health courses as well—particularly as graduate public health programs need more diversity, and UDL has been shown to increase student diversity via the flexibility inherent in this system.

## Conclusions

The field of public health needs a diverse workforce, including individuals with a variety of skills and ways of communicating. If our courses are designed in such a way that only certain types of students are able to learn and achieve their goals, then we will not be able to reach a future with the diversity public health practitioners want and need. Creating course using UDL will not only make public health classes more satisfying for both students and faculty—but it will also maximize the potential of students of different background and skillsets taking these courses and ending up in the field of public health. With enough training and institutional support, developing inclusive courses can be achieved by any faculty member. This should be a goal of all public health programs and faculties to improve the quality of education we are providing our students.

## IRB Approval

This study was evaluated by the Institutional Review Board at Boston College and was determined to be exempt, as it is a minimal risk study. IRB Protocol Number: 22.177.01e.

## Additional File

The additional file for this article can be found as follows:

10.5334/aogh.4045.s1 Supplemental Table 1. Qualitative Themes and Demonstrative Quotes.
