# Supplementary material for: Use of Universal Design for Learning Principles in a Public Health Course
Source: Ann Glob Health. 2023 Jul 21;89(1):48. doi: 10.5334/aogh.4045 (PMC10360973; doi:10.5334/aogh.4045)
Supplement: Supplemental Table 1. — Qualitative Themes and Demonstrative Quotes. [file agh-89-1-4045-s1.pdf]

Supplemental Table 1: Qualitative Themes and Demonstrative Quotes

Code	# Times Used	Sample Quote
Accessibility	4	Yeah, um I think she's very accommodating of her students and understanding that we all learn in different ways and like you can see that also in her course curriculum like rather than just heavy scientific readings, she offers podcasts and videos and YouTube videos or movies and other ways to have us interact with the material
Assignment feedback	3	And then on, the feedback front I also liked that we got, you know, detailed feedback and feedback that was helpful to know what we did right or wrong whereas I think that something I've learned in college is that professors seem to skip over the feedback part, and they're just like "Here's your grade," if they even give you your grade, so it was nice to know what I was doing right or wrong because that helps to make sure that you're being successful in the remainder of the class.
Assignment options	13	One thing that I really appreciated was that like, there was always multiple options of fulfilling your requirements because in some courses, they're very strict and very by the book that you have to do an assignment a specific way like write a paper or make a presentation or whatever, but [redacted] always gave like multiple options for what we wanted to do, and there was also like some sort of choice in what you wanted to research or look into, so.
Class organization	13	I have to agree about the Canvas--the organization of the Canvas was really nice. I know that in other classes, half the time professors don't even know where to put things. They're like, "Oh let me search to see where I put this," which is really difficult when they can't even find documents or things that we need, whereas this was, like we didn't ever have to look at the syllabus. Everything was in the order. It was very organized, everything for that topic was under the same thing, like the same umbrella, and so it wasn't like "It's in this. Oh wait, no I'm wrong. It's in this. Maybe it's in this." And so, it made it a lot easier to follow along and find things rather than when professors can't even find where things go.
Clearer connections	5	Um, we had a project this year, and it was like, you had--we had to go to the Museum of Fine Arts. And the paper that we had to write was really interesting, like I feel like I learned a lot looking back, but I just feel like, the museum trip, I--for me personally, it was a bit of stretch to make the connection, so I feel like there maybe could have been a better way to her maybe like, her help us make a connection, rather than us like go up on our own and figure it out for ourselves.
Enjoy class	3	I have to agree about the--how the class was run. I think, I'm somebody who absolutely loves to learn, in high school and all throughout my life. Like learning has always been fun, going to class has been fun. And I feel like first semester was really discouraging because learning kind of wasn't fun anymore. Like all of a sudden it became like a chore to do all these different things, and classes just weren't--it just wasn't the same. And it might have been because we were just coming out of covid, but I feel like there was a lot of discouraging class and it just wasn't fun anymore. And I legitimately was like sad the last week whenever we had our very last lesson. Like this is the first time that I've actually felt excited about learning again since we--since I've started college.
Flexibility	15	I was gonna say like I felt like this course has just been like super accessible throughout the entire year and I really appreciated that. And like, [redacted], I can definitely tell like she cares about us, like each individually as students as we go through like our--most of us are freshman, so our freshman year. And like, having like, oh Zoom class available, I know like I had covid and obviously couldn't come into class, and a lot of my other professors were like, "Sorry, there's nothing we can do. We don't record or anything," but having the Zoom option and recordings available and being available to talk over Zoom was super helpful. Just so I didn't fall behind or anything 'cause like, I was very sick so that was very helpful and I didn't put that in the survey question but I just remembered that now, so.
Inaccessible	1	And I would also say, just for me, the slides, like there's always a lot of information on them, so I think it would be helpful um to either make more of a summary on each slide, or to keep that information and just disperse amongst more slides so that it could just like be a bigger text maybe? Yeah.
Materials options	4	I really enjoyed that sometimes there would be a podcast, sometimes you have to read this article or like you could do all of that, but you could choose, y'know, do one of this or two this if I didn't have enough time to complete all of the materials presented.

Need more guidance	19	I don't know if this is just a personal thing but I know there was a timeline for our final project that I didn't really end up using... And for the other class there were like specific dates that the professor wanted drafts of our work. Um, and I think that was nice because I think it made it helpful and I knew this was there, but since there was no pressure to like, turn something in, I don't know if I--I feel like I will be hard-pressed for time in my writing. That might just be me, but maybe being more intentional about putting this timeline into place might help.
Needs more diversity	2	Oh I just had, this is an answer to the last question. I think that I learned so much valuable information from this course, and I do love that it is majority girls because it does make it a comfortable but I do think that this course should not just--it should be encouraged to like any gender so that no matter what, when you learn about maternity and motherhood and like public health, it's like really valuable to anyone, so maybe like if there was a way to kind of incentivize other people to kind of expand the diversity within the classroom, I think this is such an amazing class that...
Positive classroom environment	14	I was just gonna say that she was able to talk about like very controversial topics and current events and like, in a non-biased point of view without asserting too much opinion so regardless of what anyone feels on that topic, she would make everyone feel comfortable.
Real life application	4	I also really valued how outside of the normal curriculum, we'd learn about how that connects to the real life events going on right now, so even though we were learning about things like in the historical context, we were able to see about how like it's kind of progressed or like kind of regressed even, and then see how that associates to the things that we see in modern day.
